# Comparable prediction of breast cancer risk from a glimpse or a first impression of a mammogram

**DOI:** 10.1186/s41235-021-00339-5

**Published:** 2021-11-06

**Authors:** E. M. Raat, I. Farr, J. M. Wolfe, K. K. Evans

**Affiliations:** 1 University of York, Heslington, York, YO10 5DD UK; 2 Harvard Medical School/Brigham and Women’s Hospital, Boston, USA

**Keywords:** Gist, Radiology, Mammography, Holistic impression, Gestalt

## Abstract

Expert radiologists can discern normal from abnormal mammograms with above-chance accuracy after brief (e.g. 500 ms) exposure. They can even predict cancer risk viewing currently normal images (priors) from women who will later develop cancer. This involves a rapid, global, non-selective process called “gist extraction”. It is not yet known whether prolonged exposure can strengthen the gist signal, or if it is available solely in the early exposure. This is of particular interest for the priors that do not contain any localizable signal of abnormality. The current study compared performance with brief (500 ms) or unlimited exposure for four types of mammograms (normal, abnormal, contralateral, priors). Groups of expert radiologists and untrained observers were tested. As expected, radiologists outperformed naïve participants. Replicating prior work, they exceeded chance performance though the gist signal was weak. However, we found no consistent performance differences in radiologists or naïves between timing conditions. Exposure time neither increased nor decreased ability to identify the gist of abnormality or predict cancer risk. If gist signals are to have a place in cancer risk assessments, more efforts should be made to strengthen the signal.

## Introduction

The visual system has the remarkable capability to extract information about our environment in the proverbial blink of an eye. Within a 100 ms, humans can identify the general meaning (or “gist”) of what they are seeing (Potter, 1975). They can extract information about the scene category (Greene & Oliva, 2009) or detect the presence of certain object categories (Bacon-Macé et al., 2005). Gist extraction is a global, non-selective process, by which our visual system rapidly extracts structural and statistical regularities over the whole image to make broad categorizations of the stimulus perceived (Wolfe et al., 2011). The global, non-selective nature of the process means that the observer might be quite sure something like an animal is present but not be sure of its precise identity or location (Evans & Treisman, 2005).

This rapid gist extraction also occurs with specialized scenes like radiological images. To a non-expert, the gist of a mammogram may be nothing more than ‘this is a mammogram’. However, expert radiologists can extract a “gist of abnormality” (Evans et al., 2013a, 2013b) from a brief glimpse of, at least, some medical images. Medical experts can distinguish abnormal from normal images with above-chance accuracy after rapid exposures. Experimental studies typically use exposures of 250 to 500 ms. Reliable detection of this gist of abnormality has been found for different types of medical images, for example chest radiographs (Kundel & Nodine, 1975), prostate images (Treviño et al., 2020), cervical micrographs in cytology as well as 2D mammograms (Evans, et al., 2013a, 2013b) and 3D breast tomosynthesis (Wu et al., 2019).

While the exact perceptual features driving the extraction of the gist of abnormality are not yet known, previous research has investigated several potential factors. Breast density, which is known to be a predicting factor for breast cancer (Boyd et al., 2010; Vachon et al., 2007), cannot explain the gist signal, as it is less predictive of abnormality than gist, and shares only a small and negative correlation (*r* − 0.10–0.26), with gist ratings on the same cases (Evans et al., 2019). Similarly, global symmetry between the two breasts might facilitate gist ratings of abnormality, but is certainly not essential, as gist ratings of unilateral abnormal cases reached *d*′ of 1.16 (Evans et al., 2016), showing that while symmetry may assist distinguishing abnormal from normal cases, it is not required. On the other hand, there seems to be an important role of high spatial frequencies, as performance dropped considerably when high frequency information was removed (low-pass filtered *d*′ = 0.26). High-pass filtered images supported performance (*d*′ = 0.96) that was not markedly worse than full spectrum images (*d*′ = 1.06) (Evans et al., 2016).

One of the leading lines of evidence that the gist of abnormality is global in nature is that the gist can be detected even when no lesions are present in the presented image. Radiologists detected the gist of abnormality in patches of breast parenchyma that did not include the lesion as well as in mammograms of the breast contralateral to the one with the cancerous abnormality (Evans et al., 2016). Under these conditions, performance is reduced, but still above-chance (*d*′ = ~ 0.4 for patches, ~ 0.6 for contralateral breast). There is evidence that the global gist of abnormality is present even before any visibly actionable cancerous abnormalities are present. Radiologists distinguished between ‘abnormal’ mammograms, taken 3 years before a woman developed any actionable abnormalities and ‘normal’ mammograms from women who did not develop cancer. Accuracy was above chance with 500-ms exposure (Brennan et al., 2018; Evans et al., 2019) to these ‘prior’ images. Thus, gist of abnormality is a relatively small, but robust, global signal present in medical images, although the exact perceptual features contributing to the gist of abnormality remain a gap in the literature that requires further research.

The existence of this gist of abnormality may initially sound implausible. However, think about your first glimpse of a store. You might ask yourself if you are likely to find something that you want here. You could not do this perfectly in half a second, but neither would you be at chance. Your expertise as a consumer would allow you to register the gist of the store, even if the item you wanted was not in that first view. An expert radiologist can do something similar with a mammogram.

Unsurprisingly, gist extraction performance does not reach the performance levels obtained by experts when the stimulus remains visible during regular clinical reading. For example, a *d*′ of 1.0 was found for gist extraction of chest radiographs in 200 ms, compared with a *d*′ of 2.5 achieved during free-viewing (Kundel & Nodine, 1975). Similarly, free-viewing of a set of mammograms in a laboratory setting produced a *d*′ of 1.9 for distinguishing abnormal from normal images (Evans et al., 2013a), while 250-ms exposure produced gist performance of *d*′ ≈ 1 with 250-ms exposure (Evans, et al., 2013a, 2013b) and 1.14 after 500-ms exposure.

The increase in performance between rapid exposure and free viewing seemingly fits with two-stage detection models in medical image perception that propose to divide visual processing into an early and later stage. The first stage occurs rapidly and extracts global information about the image, not unlike gist extraction (Sheridan & Reingold, 2017). Swensson’s *Two-Stage Detection Model* asserted that a first stage filters the image and identifies features that require further examination and that a second stage carries out a search over the identified locations (Swensson, 1980). Swensson argued that medical experts have acquired perceptual mechanisms that allow them to extract and use this global information more effectively than novices. Similarly, Nodine and Kundel’s *Global-Focal Search Model* postulated that when viewing a medical image, experts obtain a global impression of the image, which constrains their subsequent search (Nodine & Kundel, 1987). The global information is extracted from an image and compared to a schema built from prior knowledge. Schemas of normal and abnormal medical images help identify potential perturbations, and focal attention is guided to these locations for further examination. In an updated version renamed the *Holistic Model,* an expert rapidly assesses an initial holistic impression in order to constrain a subsequent search-to-find process. During the search-to-find stage, holistically identified perturbations are attended foveally, while the expert also scans the image for any less salient abnormalities that were missed in the holistic stage (Kundel et al., 2007). Kundel has argued for a model of radiologist performance that has a prominent role for an “initial holistic, gestalt-like” stage of processing that is conceptually quite similar to global gist processing as we have described here (Kundel et al., 2008). However, there is an important difference between the holistic analysis of the image as Kundel et al. understand it and global gist processing as we are using it here. The holistic representation contains information used to guide attention to locations where targets are likely to be, while the gist representation is a non-localized sense that this patient might or might not have disease.

Another important difference between the Kundel account and global gist processing concerns the time frame. The holistic phase of the Kundel et al. model encompasses roughly the first full second of the reading of an image. More modern work in visual attention would envision that first second to be a mix of fast global gist processing and selective attention to a substantial number of specific objects or locations in the field (Evans et al., 2016; Wolfe et al., 2016). In the global gist experiments, stimuli were flashed briefly (typically for 500 ms or less), for the purpose of limiting volitional eye movements and attentional scrutiny of the images. This raises an interesting question; would the global gist signal continue to grow if observers had more time to look at the image? Alternatively, might the signal *only* be available if the images are briefly presented? There are phenomena that behave in this way, vanishing if the observer sees the stimuli for too long (e.g. abnormal fusion in binocular vision (Wolfe, 1983)). Accordingly, in the present experiment we compare performance of novice and expert viewers who view mammograms either for 500 ms or for as long as they like. The most interesting conditions in this experiment are those where there is no localized pathology in the image. Is the gist signal bigger, smaller, or unchanged by the ability to look longer to establish a ‘first impression’ ?.

## Methods

We compared two experiments involving rapid assessment of the same set of image stimuli using two different groups of participants: novice and expert. The first experiment presented the images very briefly for 500 ms, while the second allowed unlimited viewing time but asked the observers to make a decision on the basis of their “first impression”. The main experimental observers were two groups of medical experts in radiology, and the control group was a group of observers without medical experience (“naïves”). Prior research has shown that naïve participants, without medical training, are unable to assess if a mammogram is abnormal or not in 500 ms (Evans, et al., 2013a, 2013b). The control group allowed us to determine if naïve observers would have access to the “gist of abnormality” if they just had a bit more time. Radiologists were tested as part of the Medical Image Perception “pop-up” laboratory supported by the US NIH: National Cancer Institute at the annual meeting of the Radiological Society of North America (RSNA) in 2018 and 2019. The RSNA meeting presents a unique opportunity to test expert radiologists in numbers that are otherwise difficult to access. That opportunity comes with methodological constraints. A between-subjects design was needed as the RSNA setting did not allow for a sufficient time for ‘wash-out’ of memory for specific images between a first and second assessment of that image. Additionally, there is an inherent level of unpredictability of testing in such settings. This is reflected, for example, in the unequal numbers of observers in the two radiologist groups, one group tested in 2018, the other in 2019.

### Participants

A total of 50 participants took part in this study. A group of 11 radiologists with experience in mammography (7 female, age 32 to 65 years, 11 right-handed) participated in the no time limit condition, while 16 radiologists took part in a 500-ms time limit condition version of the experiment (9 female, age 38 to 63 years, 12 right-handed), which was part of a previously collected dataset in which spatially filtered mammograms were compared to unaltered mammograms, of which the ratings for unaltered cases formed the dataset used in the current experiment. A single group of 23 naïve observers (21 female, age 18 to 33 years old, 21 right-handed) participated both in the no time limit and the 500-ms time limit conditions.

Radiologists in this experiment were all at least at the resident level, who were currently practicing reading mammograms. They were all experienced at reading mammograms in a clinical setting, which was defined as having read at least 2000 scans in the last year. The radiologists in the no time limit group read on average 5195 scans (std 2757, range 3000 to 10,000) a year. They averaged 16 years in practice (std 9.6 years, range 4 to 30), and on average spent 63% of their time diagnosing mammograms (std 33%, range 15 to 100%) in their work. The radiologists in the rapid display time limit group read on average 5056 scans (std 3828, range 2000 to 12,000) a year, averaged 22 years in practice (std 11.9 years, range 2 to 38), and on average spent 59% of their time diagnosing mammograms (std 35%, range 15 to 100%) in their work.

The lowest value of years in practice was slightly less than used as a cut-off for expertise in some previous studies, which used a cut-off of 5 years (Chin et al., 2018; Evans et al., 2013a, 2013b), but matches the minimum years in practice used by Carrigan et al. (2018). Additionally, number of annual cases is a key determinant for good reading performance (Rawashdeh et al., 2013). A study found that readers with 2000 to 4999 annual cases outperformed those who read 1000 cases or less on malignancy detection, but were not outperformed by those with more than 5000 annual cases (Reed et al., 2010). Thus, the radiologists in this study could all be considered experienced observers of mammograms.

For the no time limit condition, radiologists were recruited during RSNA 2019. For the 500-ms time limit condition, radiologists were recruited during RSNA 2018. Naïve observers were undergraduates at the Psychology Department of the University of York (UK), participating for course credit. All participants had normal or corrected-to-normal vision. This study was approved by the Psychology Departmental Ethics Committee of the University of York, and all participants gave informed consent.

Two separate groups of radiologists were tested because a within-subject design would have required a sufficient time window between measurements to avoid memorization effects. This would not have been practical in the RSNA setting.

### Stimuli and apparatus

The 500-ms group of radiologists saw a total of 120 stimuli. The 120 stimuli were mammograms of either mediolateral oblique (MLO) or craniocaudal (CC) view of two breasts (bilateral). Of these, 60 were abnormal, composed of 20 with obvious lesions, 20 with subtle lesions and 20 mammograms acquired 2 to 3 years prior to cancer showing no visibly actionable lesions at that time. The categories obvious and subtle abnormal were based on how easily detectable the abnormality was judged to be by an experienced collaborating radiologist. The other 60 were normal mammograms that did not contain cancerous abnormalities. The 60 normal mammograms were preassigned to the three categories of abnormal, so that each performance measure was calculated between 20 abnormal and 20 normal cases. Only the trials with subtle abnormal and prior stimuli, and their pre-assigned normal stimuli were analysed in this study, since these categories were also used in the other conditions, resulting in a total of 80 trials used for analysis.

**Fig. 1:**
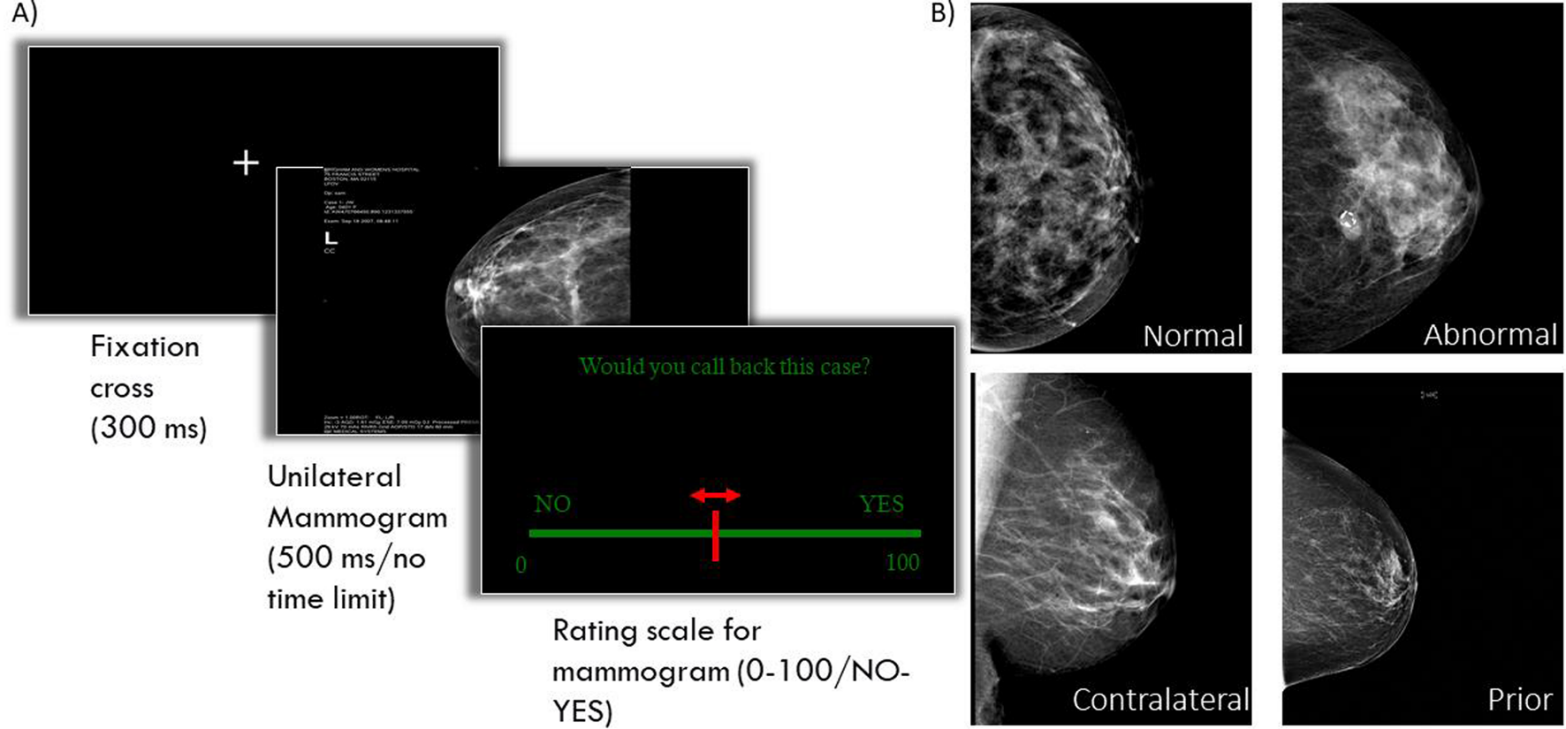
Simplified overview of the experimental procedure (**A**) and example mammograms for each of the four types used in this experiment (**B**)

The number of normal mammograms was reduced to a singular set of 20 normal cases in the no time limit condition (and both conditions for naïves) in an effort to reduce the duration of the experiment and increase ease of data collection given that in the no time limit experiment image viewing was self-paced. Thus, for the no time limit group of radiologists, and both conditions for naïves, results are based on 80 trials. The 80 stimuli were images of either MLO view or CC view of a single breast (see Fig. 1B for an example). These images were subdivided into four categories: normal mammograms of healthy women (normal), mammograms with relatively subtle cancerous abnormalities (subtle abnormal), mammograms of the breast contralateral to a breast containing a cancerous abnormality (contralateral), mammograms from women who later developed cancerous abnormalities but showed no visibly actionable lesions in these mammograms that were acquired on earlier screening (priors). Given that unilateral mammograms were presented in the no time limit experiment, we were able to add the category of contralateral images—images of a breast that did not contain a lesion but was contralateral to a breast that did contain a lesion. Thus, the no time-limit version of the experiment used a sub-selection of the cases from the time limit version, 20 of the 60 normal cases from the time limit version, the 20 subtle cases which were split to create the unilateral subtle and contralateral categories, and all 20 prior cases. Neither priors nor contralaterals contained visible cancerous abnormalities, as determined by a study radiologist. Thus, they would have been labelled as ‘normal’ in regular practice. No mask was used in the no time limit condition, since the goal was to have unlimited visual processing until the participant chose to continue to the rating screen. Due to experimental limitations, the 500-ms condition of the naïves also did not include a mask, but since this would only increase the chance of naïves detecting the gist of abnormality, this is not considered a limitation.

For the radiologists, the images were presented on a 24′ in. colour medical imaging display (1920 × 1200 pixels). For the naïve observers, the images were presented on 19.7′ in. colour monitor (1280 × 1024 px). The stimuli, themselves, were presented in the centre of the screen at a size of 800 × 1000 pixels. The experiment was run using MATLAB, utilizing the Psychophysics Toolbox 3 extensions (Brainard, 1997; Kleiner et al., 2007). All mammograms were selected from the Complex Cognitive Processing laboratory database of stimuli, which can be shared with other researchers upon request to the last author (K.K. Evans).

### Procedure

The procedures for both the no time-limit and time-limit version of the experiment were largely the same. The experiment consisted of 3 practice trials and 80 test trials (for no time-limit radiologists and for naïve observers) or 6 practice and 120 test trials (time-limited radiologists). In the practice trials, participants were familiarized with the display and rating screen, and feedback on the stimulus (normal or abnormal) was given after they confirmed their rating. On the test trials, no feedback was given. There were 20 trials for each of the abnormal types, but the time limit version for radiologists contained 60 rather than 20 normal cases (see stimuli and apparatus). Presentation order was randomized for each participant.

Each trial began with a white fixation cross presented at the centre of the screen (500 ms), followed by the mammogram being visible for either 500 ms (time-limited condition) or until the spacebar was pressed (no time-limit condition). For the time-limited experiment, the mammogram presentation was followed by presentation of a mask composed of the same breast outline, but with tissue replaced by a solid white field for 500 ms, before the rating screen was shown. No mask was used in the no time limit condition since the goal was to have unlimited visual processing until the radiologist chose to continue to the rating screen (see stimuli and apparatus). On the rating scale, participants used the mouse to move a slider to register their rating on the scale from 0 to a 100 (see Fig. 1A). Participants had to confirm their rating by pressing the spacebar, after which the next trial would start automatically. There was no masking display following the rating-scale screen.

Participants were asked to rate how certain they were that the image came from a woman with breast cancer or that the woman would develop cancer in the near future. The specific instructions given in the no time limit condition were: “You will be presented with 80 mammograms. View them for a time of your own choosing, but do not perform a detailed search of the image. Rather, focus on your first impression, your gut feeling, of the mammogram, without trying to scrutinize and search the image to localize abnormalities. Remember that 50% of the mammograms in the study contains or will develop cancer in the near future. You will then rate the mammograms on the likelihood of it containing cancer or developing it in the near future, based on your general impression, on a scale from 0, certainly no cancer, to a 100, certainly cancer present or will develop.” Instructions for the time limit condition were similar, except that it did not warn them to avoid detailed search, but instead emphasized that the image would only be visible for 500 ms.

Participants were asked to adopt a liberal rating criterion with regard to their decisions on whether a case contained or would develop cancer, while being as accurate as possible. There was no time constraint for choosing a rating in either condition, but participants were asked to report their first impression.

Different groups of radiologists participated in each of the two versions of the experiment (time limit of 500 ms and no time limit first impression). The versions were conducted a year apart. A single group of naïve participants participated in both the no time limit and the 500-ms time limit version in two different sessions, in a counterbalanced order. For naïve participants there was no masking used after the mammograms were presented in either experiment, due to the way the experiment was programmed. For naïves, each condition was tested in a separate session with at least one day and at most 1 week between sessions. Before each session, naïve participants were shown a short PowerPoint presentation to familiarize them with the concept of mammogram rating. This presentation explained how mammograms are made, how the brightness of the mammogram relates to tissue density, and common signs of abnormalities, as selected by a radiologist.

### Data analysis

The data were analysed using the framework of signal detection theory for binary classification. Given a rating, a mammogram was considered to be classified as either “abnormal” or “normal”, depending on whether the rating is higher or lower than some threshold. That classification was then compared to the ground truth. Signal detection measures were used to separately assess performance and response biases of the observer. Performance was represented by the *d*′ measure (*d*′ = *z*(true positive rate) − *z*(false positive rate)), where z denotes the inverse normal or z-transformation of the rates). In the cognitive literature, *d*′ is referred to as “sensitivity”. Unfortunately, “sensitivity” refers to the “true positive” or “hit” rate in the medical literature. We will refrain from using the term in order to avoid confusion. Response bias was measured by the criterion value, *C* (*C* = (*z*(true positive rate) + *z*(false positive rate))/− 2). A negative criterion means that the observer was more likely to label the item as abnormal, while a positive criterion means that observer was more likely to label the item as normal.

Receiver operating characteristic curves (ROC) were constructed by repeating this division of trials into proportions of true positive (hits) and false positive (false alarms) using different normal/abnormal rating cut-offs (here, 10, 20, 30, 40, 50, 60, 70, 80, and 90). The area under the curve (AUC) of an ROC, ranging from 0.0 to 1.0, represents the probability that a randomly chosen abnormal case will be rated higher than a randomly chosen normal case (Hanley & McNeil, 1982). Chance performance yields an AUC of 0.5. Higher AUCs indicate better performance in detecting the signal of cancerous abnormalities. AUCs were calculated using the trapezoid function in MATLAB.

*d*′, criterion and AUC performance measures were calculated for each of the groups and conditions. For statistical analysis, we used the *d*′ and c values derived using a rating cut-off of 50, the middle of the ROC. In all cases, false positives were derived from ratings of 20 normal images that functioned as the negative cases, using the pre-allocated subset of 20 normal cases per image type in the radiologist time limit version, or the single set of 20 in the other experiments. The true positive rates were derived separately from responses to abnormal, contralateral, and prior images. Statistical analysis was used to compare these performance measures between image types, conditions, and group. The main statistical test used was mixed ANOVA, as there were the within-group measures of image type, and the between-group factors of either group (naïve/radiologist) and/or condition (500 ms/no limit). For comparing condition effects in naïves, a repeated measures ANOVA was used as this was measured with a within-subject design. Paired t-tests, corrected for multiple comparison, were used to compare specific conditions. One-sample t-tests were used to compare performance measures to chance.

In addition, reaction time (RT) data were collected in the no time limit condition. RT was defined as the time between the appearance of the mammogram and the time when the observer confirmed their rating. Average reaction time of radiologists and naïves was compared using an independent samples t-test. Repeated measures ANOVAs were used to compare reaction times within each group between image types.

Where possible, a combination of frequentist and Bayesian statistics are reported. Bayes factors can indicate the relative strength of evidence for two theories, where BF_10_ indicates the probability of the alternative compared to the null hypothesis under the observed data. Thus, Bayesian statistics can indicate whether a non-significant *p* value from a frequentist test provides evidence towards the null hypothesis or if the evidence is insensitive (Dienes, 2014). The latter is generally considered the case with Bayes factors between 0.33 and 3. Values outside of this range provide evidence towards the null or alternative hypothesis, according to the heuristic classification scheme that was proposed by Jeffreys (1998) and is widely used to interpret Bayes factors. Bayesian statistics were calculated using the computer software JASP, version 0.14.1 (JASP-Team, 2020).

## Results

**Fig. 2:**
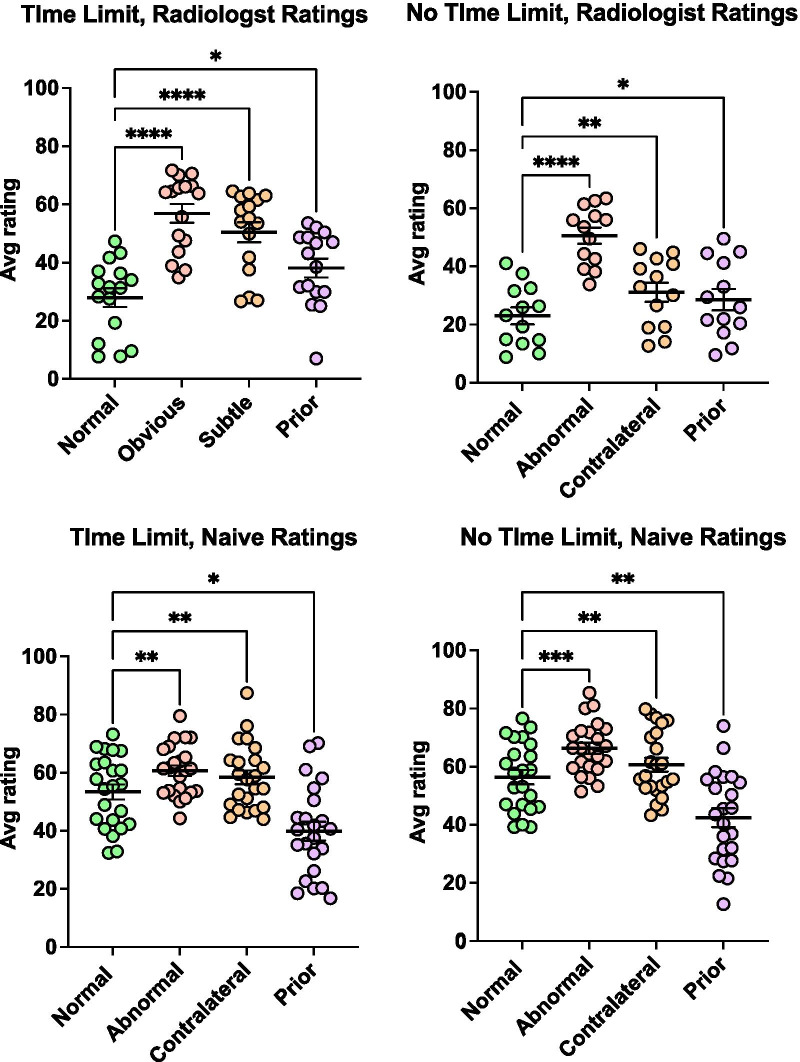
Average ratings for each observer group for each type of image. Statistical results are Dunnett’s multiple comparisons tests, comparing each type of abnormal image to the normal images

Figure 2 shows the average ratings for each observer group (Radiologist and Naïve) for each type of image. For the radiologists, Dunnett’s multiple comparisons tests show that all types of abnormal images are rated as significantly more abnormal than the normal images when viewing time was limited or unlimited (all *p* < 0.05). Interestingly, the data for the naïves also show significant differences between normal images and the other images, though the pattern of ratings is different than that seen with the radiologists. It is notable that the naïve observers rated the prior images as more normal than the normal images. This can be seen as type of artefact of stimulus selection. On returning to our image set, it appears that naïves might have used some rough assessment of density/complexity as a basis for their ratings, as the priors in this study are inadvertently systematically less dense than the normal images. The radiologists appear to be sensitive to some signal beyond density/complexity since they rate the priors as more abnormal. Since density and complexity are correlated with cancer risk, we can imagine that the radiologists took those factors into account as well. Had the images been more carefully balanced for density and complexity, it seems likely that the difference between radiologist ratings of normal and prior images would have been greater.

**Fig. 3:**
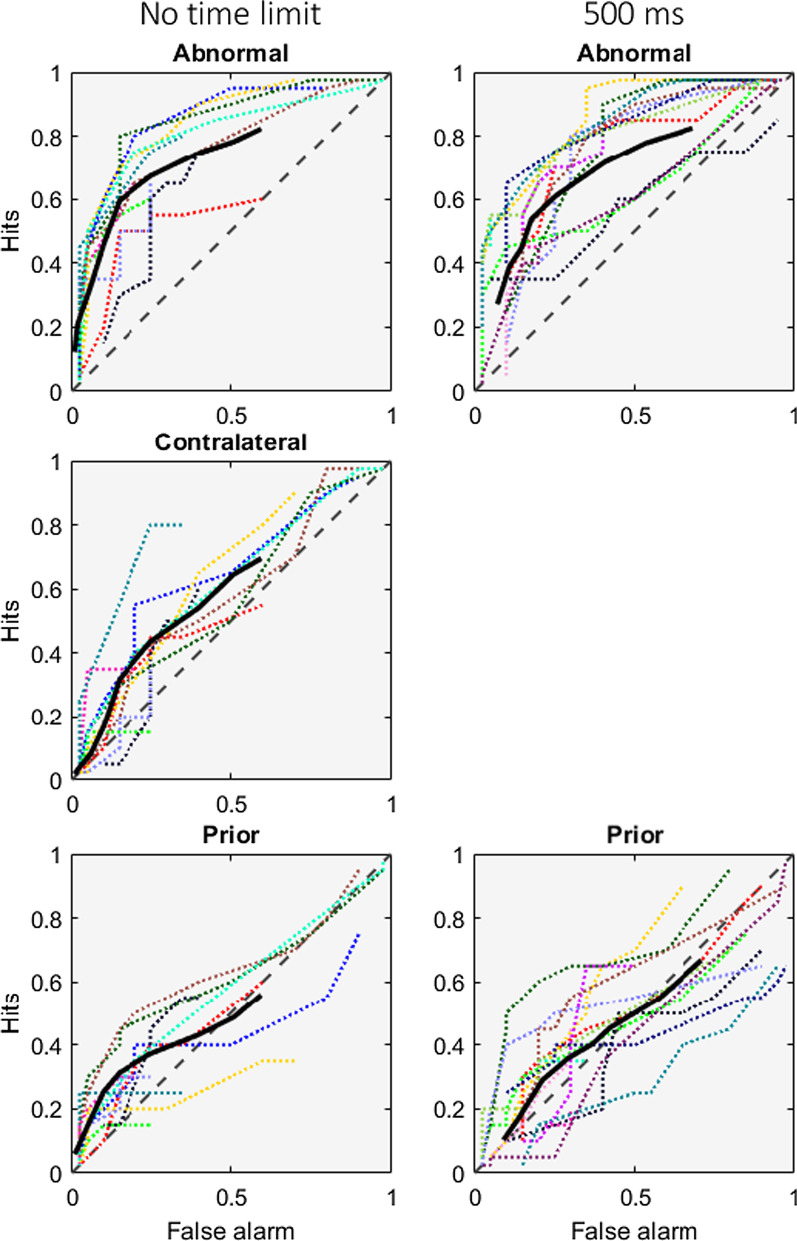
ROC curves for the radiologist groups during no time limit and 500-ms time limit conditions per image type (subtle abnormal, contralateral, priors). Each plot contains individual ROCs (coloured dotted lines) and the group mean ROC (thick black line). The dashed grey diagonal line indicates the line of no discrimination

**Fig. 4:**
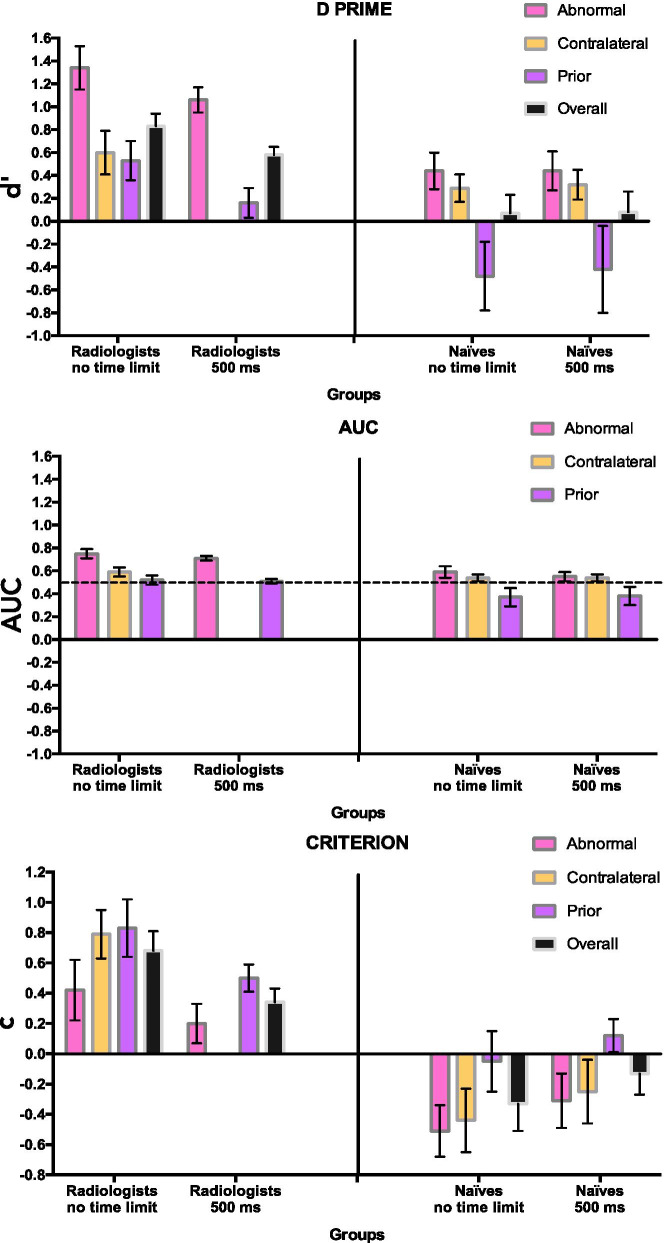
Bar graphs representing the average *d*′, AUC, and criterion (± SEM) per image comparison category (subtle abnormal, contralateral, prior) and over the total image set for the radiologists and naïves under no time limit and 500-ms time limit conditions

Turning to signal detection measures, Fig. 3 shows that the ROCs for individual radiologists mostly lie above the diagonal chance performance line, while Fig. 4 shows the average d', AUC, and criterion per image type for each group of participants. As noted, the effects for the priors are weaker than what has been seen in other studies (Brennan et al., 2018; Evans et al., 2019), but this should be seen in light of the inadvertently lower density and complexity of the prior images.

Z-transformed versions of the ROCs (zROCs) produced curved functions. zROCs are straight lines if the underlying signal and noise distributions are normal. The curved zROCs could be taken as evidence that the underlying distributions are not normal; an interesting possibility beyond the scope of the current project.

### Effect of time limit on performance in radiologists

To see how time limitations affect performance of mammography experts, 2 × 2 mixed ANOVAs were conducted on *d*′ and AUC with timing condition (no time limit, 500-ms time limit) as a between-group factor and image type (subtle abnormal, priors) as a within-group factor. As stated in the methods, no contralaterals were shown in the time limit condition for the radiologists, so these were not included in this part of the analysis. For *d*′, there was strong evidence for a main effect of image type (*F*(1,25) = 59.409, *p* = < 0.001, *ηp*^2^ = 0.704, BF_inclusion_ = 5.87e7 and moderate evidence for a main effect of timing condition (*F*(1,25) = 7.819, *p* = 0.010, *ηp*^2^ = 0.238, BF_inclusion_ = 3.828). There was no significant interaction effect (*F*(1,25) = 0.312, *p* = 0.576, *ηp*^2^ = 0.013, BF_inclusion_ = 0.727). In the AUC data, there was, again, a large main effect of image type (*F*(1,25) = 110.85, *p* = < 0.001, *ηp*^2^ = 0.816, BF_inclusion_ = 1.241e10), but no statistically significant evidence of a main effect of timing condition (*F*(1, 25) = 1.757, *p* = 0.197, *ηp*^2^ = 0.014, BF_inclusion_ = 0.613). There was no evidence for an interaction effect (*F*(1, 25) = 0.440, *p* = 0.513, *ηp*^2^ = 0.017, BF_inclusion_ = 0.392). The BF_inclusion_ for both condition and interaction effect can be classified as anecdotal evidence for *H*_0_.

Our particular interest was in whether more time allowed experts to extract more meaning from the prior images. Post hoc comparisons showed that unlimited time produced a larger *d*′ (*t*(25) = 2.796, *p* = 0.010, BF_10_ = 1.942) but not a larger AUC (*t*(25) = 1.325, *p* = 0.197, BF_10_ = 0.378) on average, and the Bayes Factor for the *d*′ difference shows only anecdotal evidence. The combination of non-significant effect on AUC and anecdotal Bayes Factor for *d*′ suggest that this might not be a true difference. Looking at Fig. 3, it is clear that performance is above chance in both conditions but that the variability between observers makes it hard to determine if unlimited time improves performance. Certainly, unlimited time does not produce a massive improvement.

Turning to the criterion, there was a main effect of image type (*F*(1,25) = 52.290, *p* = < 0.001, *ηp*^2^ = 0.677, BF_inclusion_ = 322.440). There was no evidence of main effect of timing condition (*F*(1,25) = 3.247, *p* = 0.084, *ηp*^2^ = 0.115, BF_inclusion_ = 1.331) or an interaction effect (*F*(1, 25) = 0.405, *p* = 0.530, *ηp*^2^ = 0.016, BF_inclusion_ = 0.423). Criterion was significantly higher for priors than subtle abnormal cases (mean difference = 0.345, *p* = < 0.001, BF_10,U_ = 416.754).

These findings showed some indication that additional time might improve performance of radiologists on detecting future abnormality in the priors, but this effect was inconsistent, as it was observed for *d*′ but not AUC. Additionally, for *d*′, the Bayesian statistics suggested only anecdotal evidence, further weakening the evidence. Overall, our results show no clear evidence of an advantage of either time condition.

### Effect of time limit on performance in naïves

Overall performance as measured by *d*′ of the naïve participants was not significantly different from zero, as measured by a one sample t-test for the 500 ms (*t*(22) = 1.330, *p* = 0.196, BF_10_ = 0.308) and the no time limit (*t*(22) = 1.309, *p* = 0.204, BF_10_ = 0.301) condition. This is in line with previous findings and suggests that overall, the naïve participants could not detect the gist of abnormality in abnormal, contralateral, and prior images with above-chance accuracy, even without a time limit, emphasizing the necessity for perceptual expertise. More detailed analysis of the performance of naïves is available in “Appendix”.

### Effect of image type and expertise on reaction times

To investigate whether observers spend longer judging certain cases we examined reaction times under no time limit conditions. Radiologists had an average reaction time of 5526 ms ± 1884, while naïves had an average reaction time of 4213 ± 942. Radiologists' RTs were higher for each image type (Table 1). The difference between groups was significant (independent samples t-test, mean difference = 1298 ms, *t*(34) = 2.6, *p* = 0.014, *d* = 0.9) probably indicating that experts had more to think about when looking at an image.

**Table 1:** Average reaction time in milliseconds for naïves (*n* = 23) and radiologists (*n* = 11) during no time limit conditions, per image type (± 95% CI)

	Normal	Subtle abnormal	Contralateral	Priors	Overall
Naïves	4263 ± 978	4132 ± 929	4085 ± 911	4377 ± 942	4213 ± 942
Radiologists	5537 ± 1661	6162 ± 2261	5501 ± 1715	4846 ± 1735	5526 ± 1884

For naïves, a one-way RM-ANOVA on image type (normal, subtle, contralateral, prior) showed no significant effect of image type (*F*(3,66) = 1.49, *p* = 0.226) on reaction time, which was also supported by the Bayesian RM-ANOVA with a BF_10_ of 0.285 indicating moderate evidence towards this null effect. On the other hand, for radiologists, a one-way RM-ANOVA on image type (normal, subtle, contralateral, prior) showed a significant main effect of image type (*F*(3,36) = 8.80, *p* < 0.001), which was also strongly supported by the Bayesian RM-ANOVA with a BF_10_ of 139.55 indicating extreme evidence towards this main effect. Frequentist post hoc tests with Holm correction for multiple comparisons showed that responses were significantly slower for normal (*p* = 0.048) and subtle (*p* < 0.001) than prior cases, which was supported by the Bayesian post hoc tests with moderate evidence for normal and prior (BF_10, u_ = 6.83) and very strong evidence for subtle and prior (BF_10, u_ = 38.33). The frequentist post hoc tests trended towards faster responses to normal than subtle cases (*p* = 0.052), faster responses to contralateral than subtle cases (*p* = 0.052), and faster responses to prior than contralateral cases (*p* = 0.052). Among these trends, Bayesian post hoc tests showed strong evidence for a difference between normal and subtle (BF_10, u_ = 17.27), but only anecdotal evidence for subtle and contralateral (BF_10, u_ = 1.74) and contralateral and prior cases (BF_10, u_ = 1.77). The strong Bayes factor for normal and subtle cases suggests that this is a true effect, while there is only anecdotal evidence for the other two trends. Overall, reaction times differed significantly between image types, with faster responses to prior than both subtly abnormal and normal cases, and faster responses to normal than subtly abnormal cases.

## Discussion

In previous work, we and our colleagues have found that with 500 ms of viewing time, expert radiologists can use a global gist of abnormality signal to classify normal from unilateral abnormal mammograms. More strikingly, we found that that this gist of abnormality can be detected in contralateral and prior-abnormal mammograms (Brennan et al., 2018; Evans et al., 2016, 2019). In the present study, we asked if that gist signal would be markedly stronger if experts could scrutinize the image or, alternatively, if the brief exposure was required, with any gist signal being hidden by sustained exposure. In fact, the data did not show either of these effects. The existence of a gist signal was replicated but there were no dramatic effects of exposure duration.

The data from naïve participants continue to show that detection of the gist of abnormality requires expertise. As expected, performance of naïve participants was not significantly different from chance in either the no time limit or the 500-ms condition. The prior images were judged to be more normal than the actual normal images; a result that seems to reflect lower density particular in the prior images we used. This finding fits with the previous reports of at-chance performance of naïves with rapid exposure (Evans, et al., 2013a, 2013b), and also shows that more time does not enable naïves to access an accurate first impression to perform above chance. Thus, radiologists possess an ability that allows them to accurately perceive the gist of abnormality in mammograms that does not seem to be present in naïve participants, regardless of time constraints.

A central question for this study was whether the gist of abnormality would still be available to expert observers when the stimulus was not flashed but was available until response. It could have been that with longer exposures, a transient gist signal becomes diluted or cancelled by more sustained processes. Alternatively, it could be that experts could exploit the gist signal more effectively given more time. The data show that experts continue to perform at above chance levels with unlimited time, with some evidence that *d*′ was higher in the no time limit condition, but since this was not replicated in the AUC data there was no consistent evidence for improvement in performance without time-limited exposure. In thinking about a possible clinical role for gist, this is something of a disappointment. The gist signal for prior images is reliable but weak. The possible use of such a signal as imaging biomarker would be strengthened if conditions could be found that produced a more robust signal.

For the abnormal images, the images that contained visible lesions, our experts seem to have followed our instructions not to scrutinize the images. While this is a difficult instruction to verify, it is certainly the case that our average total reaction time of 5.53 ± 1.88 s is markedly lower than any normal interpretation times in the clinic (e.g. 128 to 138 s for routine screening examinations of digital mammography (Berns et al., 2006; Kuzmiak et al., 2010)) or in the laboratory (e.g. average reading time per 2D mammography case was 33 s in a screening-like condition (10% prevalence) of an archival set by 3 radiologists (Bernardi et al., 2012). Those cases included multiple images but even so, 5.5 s for one image would be hasty under normal instructions. In a two-decision stage study on bilateral cases, the initial normal/abnormal distinction took 23 s on average, followed by an additional 39 s to localize any abnormalities in the final decision phase (Nodine et al., 2002). Thus, in the current no time-limit condition, radiologists were relatively fast in making their decision, supporting the notion that they were indeed using a first impression rather than a detailed examination to inform their rating.

Response times of radiologists were significantly affected by image type, with faster responses to priors than normal (+ 704 ms) or subtle abnormal cases (+ 1323 ms). Additionally, responses to normal cases were faster than subtle abnormal cases (+ 619 ms). These differences suggest that the presence of a local abnormality increased reaction times. One could speculate that once there was no time limit the experts started looking for a visibly localizable signal of abnormality rather than a global perturbation of the parenchyma. Basing one’s decision on detection of a visible local lesion is in line with clinical practice to reduce false alarms, cognisant of low prevalence of breast cancer in screening population. In contrast, the possibility to search for local lesions is not present when the image is flashed for 500 ms, meaning the radiologist must heavily rely on their global gist impression. This might make it easier to focus on information conveyed by global, non-localizable signals of abnormality during the first impression and thus maybe a more optimal approach when aiming to develop a method for early-stage triage to identify at-risk women for more frequent screening. On the other hand, this could also result in missing possibly critical information present in the global parenchymal perturbation absent of a visible lesion. However, as our data showed no consistent changes in either performance or criterion, any changes in rating strategy between the conditions did not significantly affect radiologist ratings in our paradigm. This might be due to the mix of mammograms containing visibly actionable lesions and mammograms without visible abnormalities (contralateral, priors), which could prevent the radiologists from shifting to a strategy aimed at detecting the gist of abnormality in these more ambiguous cases. It might be interesting to repeat the no time limit condition in a new experiment using a test set composed exclusively of normal images and abnormal prior images. Such a set would lack any localizable abnormalities. With such a set, one could, give readers the information that in this stack of 100 images, 50 came from women who would develop cancer within 3 years. Readers could be asked to sort the images into normal and abnormal categories, taking as much time as they cared to. Readers could be given case-by-case feedback after each response. Perhaps these conditions would produce stronger evidence of sensitivity to the gist of abnormality.

One additional consideration is that rating cases based on either a glimpse or a first impression is not a typical behaviour for radiologists. It is possible that further training with the task for possible triage of cases could improve their performance in gist and/or first impression ratings. For example, they might become more accustomed to suppressing their inclination to perform a detailed examination without a time limit or become more attuned to their first impression in both conditions. Or, if feedback is given, they might be able to further fine-tune their gist categorization, although this might require intensive training to affect perceptual processing. These options could be explored in future experiments using training paradigms.

## Conclusion

In the present study, there was no clear evidence of additional additive benefit to the overall global impression of an image with no time limit exposure without search. Medical experts show the same overall performance detecting abnormalities in mammograms whether they use the global gist signal based on rapid viewing or using their first impression assessment with no time constrained viewing. Medical experts are not more sensitive to the signal of cancer with more time following first impression rather than gist but maintain a conservative criterion for images with no locally visible lesions.

In conclusion, it remains interesting that experts are sensitive to a global signal of abnormality that can be detected in images acquired years before the cancer produces a localized sign in the images. However, this signal remains small and was not meaningfully enhanced by removing the viewing time limit when rating a mixed set of cases in a laboratory setting. Thus, if this signal is to have some clinical utility, it is worth continuing efforts to enhance that signal by for example image enhancement.

## Data Availability

The datasets generated and analysed during the current study are available on our OSF repository, https://doi.org/10.17605/OSF.IO/5NWP8. These data are available under Creative Commons Attribution-NonCommercial-ShareAlike 2.0 UK: England and Wales (CC BY-NC-SA 2.0 UK).

## Appendix: Detailed effect of time limit on performance in naïves

The performance of naïve observers was characterized by lower *d*′ values, and AUC values close to chance (0.5) in both conditions. Following the very low ratings for priors, shown in Fig. 2, we performed one-sample t-tests to further investigate this, which showed that naïve observers’ *d*′ actually falls below 0 and the AUCs is less than 0.5. This is the case for the 500 ms presentation (AUC: *t*(22) = − 2.774, *p* = 0.011, BF_10_ = 4.51; *d*: *t*(22) = -2.139, *p* = 0.044, BF_10_ = 1.47) and no time limit conditions (AUC: t(22) = -3.233, *p* = 0.004, BF_10_ = 11.09; *d*: *t*(22) = -3.06, *p* = 0.006, BF_10_ = 7.85). However, in the 500-ms condition, the Bayes factor for *d*′ provides only anecdotal evidence towards a significantly negative *d*′ prime in that condition.

As with the radiologists, the naïve observer data for *d*′, AUC, and criterion were analysed in separate 2 × 3 repeated measures ANOVA with condition (no time limit, 500-ms time limit) and image type (normal-abnormal, normal-contralateral, normal-priors) as factors. There was a main effect of image type for both *d*′ (*F*(1.26, 27.78) = 26.18, *p* = < 0.001, *ηp*^2^ = 0.543; BF_inclusion_ across matched models = 3.75e12) and AUC (*F*(1.23,43.01) = 27.808, *p* < 0.001, *ηp*^2^ = 0.558, BF_inclusion_ across matched models = 3.75e12), but no evidence of a main effect of timing condition. Nor were there significant interaction effects. In fact, the BF_inclusion_ across matched models for condition was 0.187 (*d*′) and 0.195 (AUC), both providing moderate evidence for the null hypothesis of no main effect of timing condition.

For criterion, there was evidence of a main effect of image type (*F*(1.26, 27.78) = 26.18, *p* = < 0.001, *ηp*^2^ = 0.543, BF_inclusion_ across matched models = 2.97e7), and a main effect of timing condition (*F*(1.16, 22.00) = 4.67 *p* = 0.042, *ηp*^2^ = 0.175, BF_inclusion_ across matched models = 30.55). A pairwise comparison showed that criterion was higher (more conservative) in the 500-ms time limit conditions (mean difference = 0.184, *p* = 0.042). Pairwise comparisons of image types showed that criterion was significantly higher when rating priors than abnormal (mean difference = 0.45, *p* = < 0.001) and contralaterals (mean difference = 0.38, *p* = < 0.001). This analysis suggests that removal of time limit had no effect on performance in naïves aside from making their ratings more conservative.

## Abbreviations

AUC: Area under the curve
BF: Bayes factor
